# Progress in Medicine

**Published:** 1898-11-19

**Authors:** 


### Progress in Medicine.

### DISEASES OF THE BLOOD.

Walter K. Hunter,[1] writing upon the white corpuscles, endeavours to harmonise the classifications made by Metschnikoff, Ehrlich, Kanthack, and Gulland. (*a*) The first class of white cells recognised by all writers is the coarsely granular eosinophile, of a diameter of 10 micro-millemetres. The nucleus takes the form of a bent bar or horseshoe. These cells form only about 3 per cent. of the leucocytes in normal blood, but abound in bone marrow and in spleno-medullary leukæmia. (*b*) The next is the finely granular oxyphile, or polynuclear cell, forming 75 per cent. of normal leucocytes. Active in movement, phagocytic in habit, and measuring 9$\mu$ in diameter, it has a nucleus of irregular shape, often divided into several portions. (*c*) The lymphocyte measures only 6$\mu$ across, and shows a deeply staining nucleus which fills up almost the entire cell. It forms about 20 per cent. of the normal white cells, though this is increased after food. (*d*) The hyaline cell, or macrophagocyte, is a hyaline or finely granular basophile cell, with rounded nucleus, measuring 10$\mu$ across, and forming 2 per cent. of the cells. It is phagocytic and motile. A very small cell, with trilobed or spherical nucleus and clear plasma, is also described by Kanthack and Hardy. (*e*) The coarsely granular basophile, *mastzellen* or myelocyte, is a large cell, measuring 12$\mu$ or more. The plasma is filled with deeply staining granules, but the nucleus stains feebly, except in certain cases. It is rarely found in the blood, save in spleno-medullary leukæmia. Hunter has at times found them also staining with eosin, sometimes having a nucleus filling nearly the whole cell, and faintly stained, and sometimes having a nearly hyaline plasma. However, they take up, he says, the eosin but faintly, and can, therefore, be distinguished from the coarsely-granular true (*a*) eosinophiles. In all these cells there is a reticular nucleus, and a plasma also crossed by a fine network, the knots of which form the granules. These knots, or microsomes, and the network, or mitoma, are most developed where the cell is an actively moving one. Gulland thinks that all kinds of cells are developed from lymphocytes, and that these differences are due to the work they do and their surroundings. On the other hand, Kanthack and Hardy regard each form as genetically distinct, except that the hyaline is the mature form of the lymphocyte. As to their functions, Hunter holds that the lymphocytes (*c*) and the small, finely-granular cell of Kanthack are merely immature forms, while the polynuclear, the coarse-grained eosinophile and the hyaline (*b.a.d.*) are employed in inflammatory processes. The eosinophile is said to go first to the attack of any invading microbes, and to paralyse them by excreting a poison. The hyaline and polynuclear next follow and devour the microbes paralysed by its action.

It is well known that the number of corpuscles found in cases of anæmia is at times apparently high, and that in any individual it varies rapidly with the state of food and exercise. This has been said to depend on the degree of dilution or the total volume of the blood at the moment. Mary P. Jacobi[2] records several cases of definite anæmia in neurasthenic persons where the count was abnormally high. Massage, diarrhœa, exercise, sweating, and anything which raises the tension by vaso-motor constriction increases the number by concentration of the blood. Thus she points out we are liable to be deceived by the apparent richness of the blood when vascular spasm exists. In one of her cases a count of eight millions occurred between two of three millions each in the same patient. The sphygmograph may help us to discount such discrepancies. George Oliver[3] prefers to judge the state of the blood by the ratio of hæmoglobin to corpuscles. In both these there are daily and hourly fluctuations from the transfer of water to and from the blood, and other causes. We should, therefore, test the blood at the same time after meals, and at the same hour of each day, to obtain a just comparison, and we must allow a variation of 10 degrees from the standard as normal. Oliver's ratio (hæmoglobin to the number of corpuscles) may be normal in cases where both figures are very low. In chlorosis both numbers are extremely low, though the volume of the corpuscles is higher than that of the hæmoglobin, and their number is really higher still because they are of unusually small size. It has been supposed that the blood is greatly diluted in this disease, and that its total volume is above the normal, but of this we have no proof. Both the measuring of the hæmoglobin and of the volume of the corpuscles have been rendered very quick and simple operations by two little instruments invented recently by Oliver, which he describes. Heiman's paper on current methods of blood examination[4] lays stress on the value of the hæmatocrite for measuring the volume of the cells. Four minutes, with 6,000 revolutions a minute, suffices to show the volume in normal blood, which is 50 per cent. The specific gravity of the blood can be found by mixing chloroform and benzol in such proportions that the specific gravity of the mixture is 1,059. A drop of blood is then added. If it sinks, more chloroform is added; if it rises, benzol is poured in. When it remains in the centre, the specific gravity of the mixture, which is equal to that of the blood, is taken at once. For staining, he recommends that the blood be smeared on a glass slide, dried, fixed by formal in vapour, or by soaking in equal parts of ether and alcohol for 15 minutes, placed in eosin of 1 per cent. strength for five minutes and then washed. A double stain can be given by placing in methylene blue for two minutes, and again washing. By this method, the nuclei of the leucocytes and the plasmode of malaria are easily defined, for exact study. We may here refer to a mode of estimating the amount of iron in blood and other fluids by the colour produced by thiocyanates when added to persalts of iron. The procedure was described by W. Mackie in the *Lancet* of January 22nd, but the details cannot be given in an abstract.

**Hæmophilia.**—A. Fry[5] reports the results of treatment by injections of serum. Feltz and Pigot had previously treated purpura by injecting artificial serum with success, and the writer used horse serum in three cases of hæmophilia. One patient had, at the time of writing, received 300cc. of serum, divided into several injections. His joint troubles had disappeared, and his finger, when cut accidentally, no longer bled severely. The other two cases had received 200 and 90cc. each, and had shown improvement in all their symptoms. The puncture for the subcutaneous injections only bled on the first occasion. Albumen did not appear in the urine after the use of the serum. It may be noted here that Wright, of Netley, has recommended the use of chloride of calcium internally in some of these cases, together with inhalations of carbonic acid, and the external application of it, with or without ferments obtained by mincing normal gland tissue. Another writer has recently advised dropping a little normal blood on to a wound in a hæmophiliac person to cause clotting. Petrone[6] finds that the plaques of Bizzozero are the agents in preventing undue coagulation of the blood, while coagulation itself depends on the action of hæmoglobin and the ferment on the fibrinogen.

M. Lucien Roques[7] showed a case of paroxysmal hæmoglobinuria, the attacks of which were produced or brought on by any chill, and accompanied by factitious urticaria. Now these patients occasionally show simple urticaria, but factitious urticaria in normal persons is prevented by cold. The combination of the two affections has not been previously observed. Rolleston describes a case showing symptoms of both Raynaud's disease and erythro-melalgia. It was aggravated by cold, and the skin was red and hypersensitive. The patient had suffered from frost-bite and chilblain, and the finger-tips were clubbed. This patient was a strong young navvy, and complained of severe pain, throbbing and swelling in his hands and feet in cold weather (*Lancet*, March 19th).

**Leukemia.**—Musser and Sailer[8] record an instance of the splenic type, in which the lymphatic glands were also affected. The white corpuscles finally reached 382,100. There were three forms of mono-nuclear cells, some very large with faintly staining nuclei. The three varieties amounted to 66 per cent., the lymphocytes to only eight, and the polynuclear to 24. In the lymph glands were enormous cells of the latter type.

Rose Bradford[9] remarks that it is specially characteristic of leukemia that the red cells are diminished, the white usually greatly increased, a number of cells appear which are not normally present, and the proportion of the numbers of the various white cells is altered. In one form the lymphocytes are greatly increased, in another myelocytes are present, though never seen in health. These blood types usually correspond to clinical forms, in which the glands and the spleen respectively are enlarged, but the correspondence is not invariable. Rarely we find the bone marrow alone, among the solid tissues, to be the seat of change. It is worth remembering that patients suffering from leukemia often have a rosy complexion, and no appearance of anæmia. They may complain merely of pain in the side, weakness, or asthmatic attacks. We may be deceived by cases where there is just at the time no increase of white corpuscles, or by simple leucocytosis, where an increase of polynuclear corpuscles is caused by an abscess or cancer. The writer entirely disbelieves in the value of oxygen inhalations. W. Ewart[10] has employed inhalations of carbonic acid, together with a little oxygen, for three, seven, or ten minutes several times a day, and speaks favourably of the trials he has made of it in this disease. An instance of acute leukemia, of which only 40 cases have been hitherto reported, occurred under the care of Professor W. H. Thomson.[11] The patient had been ill for six weeks after an attack of sore throat, and died ten days after admission. The leucocytes showed lymphocyte forms to the amount of 17 per cent., myelocytes 32, polynuclear cells 48, and eosinophiles 3 per cent. Nucleated red corpuscles were also to be seen. It is remarkable that no enlargement of the spleen or glands could be detected during life, but anæmia, pyrexia, and a moderate increase of the leucocytes could alone be found. Necrotic changes in the liver and degeneration of the marrow were noticeable, and the writer was struck by the likeness to the results of toxic infection. Jawin[12] observed the course of splenic anæmia in a man who had suffered from some ill-defined infective disease. There were great numbers of nucleated red cells, but the white cells were never increased. The spleen and liver were enlarged, and the glands slightly so. Besides the poikilocytosis, a few mast cells were noticeable.

**Addison's Disease.**—Swale Vincent[13] remarks that the supra-renal capsule is a double glandular organ, the medulla being derived from the sympathetic nervous system, and the cortex from germ epithelium, the two being as distinct as possible. Injections of supra-renal extract cause great contraction of the vessels of the body and a rise of blood pressure, still it is possible that it normally acts by getting rid of the waste products of metabolism. Vincent finds that the capsules have poisonous effects when injected in large quantities, and that they are the only part of the body which has. This poison appears to exist only in the medulla, and acts both on the central nervous system and on the muscles themselves. The symptoms of Addison's disease include pigmentation and extreme muscular prostration upon exertion. Feeding with supra-renal capsules has utterly failed, but subcutaneous injections of an extract are more promising, and may be made intravenous when the case is urgent. In heart failure from chloroform it has been proposed to inject supra-renal extract into the heart itself, and in purpura and pernicious anæmia it may be tried with caution.

The active principle of the medulla seems to be closely connected with piperidine, and also to contain a chromogen allied to tannin. Becliere[14] showed a patient who had lost all the symptoms of Addison's disease under treatment by extract of calves' supra-renal, and had remained well for three years. Gaillard, Widal, and Hayem had also treated patients in the same way. One case failed entirely, two others showed a great temporary gain of strength. Mankovsky[15] prepares the extract from cows, and recommends it in heart failure where collapse is threatened. He refers the rise of blood pressure to a stimulation of the peripheral sympathetic system. Addison's disease may occur without any pigmentation, as in a case reported by T. Anderson (*Lancet*, June 18th), where exhaustion, emaciation, vomiting, and a rapid pulse led to a fatal result. There was occasional abdominal pain, and Ebstein[16] has recorded several instances of similar symptoms without pigmentation.

[1] Glasgow Med. Jour., May. [2] Med. Rec., June 25. [3] Clin. Jour., Feb. 9. [4] New York Med. Jour., May 7. [5] Med. Rec., July 23. [6] Amer. Med. and Surg. Bull., Mar. 25. [7] Lancet, June 18. [8] Med. Chron., Feb. [9] Clinic Jour., June 1. [10] Brit. Med. Jour., July 23. [11] Med. Rec. Mar. 5. [12] Brit. Med. Jour., Mar. 5. [13] Birmingham Med. Review, April. [14] Lancet, Mar. 12. [15] Brit. Med. Jour., May [illegible]2. [16] Amer. Jour. Med. Sci., Feb.

